# Foodborne Illness Complaint Systems Detect, and Restaurant Inspection Programs Prevent Restaurant-Associated Foodborne Illness Outbreaks

**DOI:** 10.1089/fpd.2023.0086

**Published:** 2024-02-05

**Authors:** Thuy N. Kim, Alexandra R. Edmundson, Craig W. Hedberg

**Affiliations:** Division of Environmental Health Sciences, University of Minnesota School of Public Health, Minneapolis, Minnesota, USA.

**Keywords:** foodborne illness outbreak, foodborne illness surveillance, restaurant inspection, foodborne illness prevention, restaurant outbreaks

## Abstract

Restaurants are important settings for foodborne illness transmission. Environmental health agencies routinely inspect restaurants to assess compliance with food safety regulations. They also evaluate foodborne illness complaints from consumers to detect potential outbreaks of foodborne illness. Local environmental health agencies were surveyed to identify methods used to conduct surveillance for consumer complaints of foodborne illness, link them to inspection grading and disclosure practices, and evaluate the association between these practices and the number of foodborne illness outbreaks in restaurant settings reported to the Centers for Disease Control and Prevention. We developed a novel framework for assessing the effectiveness of restaurant inspection grading and disclosure of inspection results while accounting for any biases introduced by surveillance factors that affect outbreak detection. Our findings showed the importance of routine restaurant inspection grading and disclosure practices as prevention measures and having a centralized database to manage consumer complaints as a useful surveillance tool for detecting outbreaks. Improving consumer complaint system structure and management can bolster outbreak detection and maximize limited public health resources while increasing the efficiency of complaint-based surveillance.

## Introduction

Advances in laboratory diagnostics (Kubota et al., 2019; Ribot et al., 2019) and epidemiologic methods (Jervis et al., 2019; White et al., 2021) have improved the ability of public health agencies to detect, investigate, and prevent foodborne illness (Scharff et al., 2016). These advances have led to increased detection of transmission occurring in restaurant settings, which are known as important settings for foodborne illness transmission. Of the 841 foodborne illness outbreaks reported to the Centers for Disease Control and Prevention (CDC) in 2017, 489 (58%) were attributed to food prepared in restaurant settings, with 366 (48%) specifically from sit-down dining restaurants (CDC, 2019).

While interventions implemented to control the COVID-19 pandemic led to 10–41% decline in pathogens transmitted commonly through food in 2020, compared with the preceding 3 years, the number of restaurant-associated outbreaks declined by 70% (CDC, 2022; Ray et al., 2021). This occurred in the context of a 15% drop in expenditure for food consumed outside the home (U.S. Department of Agriculture, 2023). The impact that pandemic-initiated restaurant restrictions had on the reduction in outbreaks further illustrates the close relationship between interventions implemented in restaurants and foodborne illness.

There are two primary sources of surveillance for detecting outbreaks of foodborne illness: pathogen specific and complaint based (Council to Improve Foodborne Outbreak Response, 2019). Pathogen-specific surveillance is primarily responsible for detecting outbreaks caused by agents such as *Salmonella* and Shiga toxin–producing *Escherichia coli*, which are detected through clinical laboratory testing.

Restaurants are frequently identified as settings for subclusters of cases in outbreaks associated with distribution of contaminated food commodities, often identified through pathogen-specific surveillance. However, most outbreaks in restaurants are detected through surveillance of complaints from consumers patronizing the establishment (Hedberg et al., 2008). Thus, the effectiveness of consumer complaint surveillance by local health agencies may influence the number of foodborne outbreaks the agency is likely to detect.

A survey of local public health agencies that conducted restaurant inspections (*n* = 140) in 2020 found that agencies that conducted grading of restaurant inspections and routinely disclosed the results to the public reported fewer foodborne illness outbreaks compared with agencies that did not grade or disclose inspection results (Kim et al., 2022; Kim et al., 2021). These findings suggested a potential protective effect of these practices on foodborne illness.

However, there was also a positive association between the number of foodborne illness complaints received by agencies in the study and the number of outbreaks detected. A lack of detail on how agencies conducted surveillance of consumer complaints precluded a more comprehensive evaluation of the relationships between potential prevention effects of grading and disclosure practices and the effectiveness of outbreak detection through consumer complaint surveillance.

The objectives of this study were to (1) identify methods that restaurant inspection agencies use to conduct surveillance of consumer complaints of foodborne illness; (2) link complaint surveillance methods to inspection grading and disclosure practices; and (3) evaluate the association between complaint system methods of restaurant inspection agencies and the number of foodborne illness outbreaks in restaurant settings reported to the CDC Foodborne Disease Outbreak Surveillance System (FDOSS) (CDC, 2018), while adjusting for the effects of restaurant grading and disclosure as prevention measures.

## Materials and Methods

Respondents to a previous Restaurant Grading Survey (Kim et al., 2021) were contacted through e-mail and phone to obtain information about how they conducted surveillance of consumer complaints of foodborne illness reporting food service establishments in their jurisdiction. This new survey (Complaint System Survey) was launched on March 11, 2022, and closed on January 2, 2023.

We matched respondent agencies by jurisdiction to the exposure locations for restaurant-associated outbreaks reported to the national FDOSS (CDC, 2018), as previously described (Kim et al., 2022). Briefly, these FDOSS data, extracted on November 18, 2019, included foodborne outbreaks where food was prepared in a restaurant setting within the jurisdiction of the agency and for which the first illness occurred between January 1, 2016, and December 31, 2018.

From the Restaurant Grading Survey, we used the following variables: number of restaurants, cumulative average number of complaints (2016–2018), average number of routine inspections performed per year, disclosure practices (online, point of service, or none), and grading practices (numerical score, letter grading, or none). From the Complaint System Survey, we examined the following variables: having a complaint system, operating a centralized complaint database, and collection of complainant's food history at initial complaint intake.

Analysis for this study included the subset of agencies that had a complaint system (*n* = 70). Those without a system represented only 9% of respondents and, of those, only one agency had a recorded outbreak in FDOSS. We obtained the number of restaurant outbreaks from FDOSS.

### Model selection and statistical methods

The outcome for this study was the cumulative count of restaurant outbreaks where the exposure occurred within the jurisdiction of the responding agencies for the 3-year study period of 2016–2018. Our overarching theoretical assumption was that foodborne outbreaks occur annually in every jurisdiction whether they are detected and reported or not. As such, we did not allow a count of zero outbreaks for any agency.

We compared Akaike information criterion and Bayesian information criterion values (Burnham and Anderson, 2004; Kuha, 2004) for general and zero-truncated Poisson and negative binomial regression models to determine goodness of fit. Based on comparison of these goodness-of-fit values between models, and a statistically significant likelihood ratio test (LRT) based on chi-square distribution, we chose a zero-truncated negative binomial regression model (Hardin and Hilbe, 2015) to calculate adjusted incidence rate ratios (IRRs).

The level of significance was set at alpha = 0.05. Analyses were conducted using SAS 9.4 (SAS Institute, Cary, NC). The University of Minnesota Institutional Review Board deemed this study as not human research.

We concluded that there was no clustering in our dataset after conducting a sensitivity analysis of the final model. The sensitivity analysis consisted of adding a fixed-effect variable for the states in which the responding agency was located to the final adjusted model. Compared with the model without the state variable, the model including state resulted in minor magnitude changes, but the trend and statistical significance of the other variables remained the same.

No overdispersion was evident in the final model, as indicated by the LRT of alpha (which was not significant) and an overdispersion parameter, which was less than zero.

### Predictor selection

Because the effects of restaurant grading and disclosure might be mediated by complaint surveillance effectiveness, adjustment for complaint surveillance characteristics is necessary to more accurately estimate the direct effect of grading and disclosure on the number of restaurant outbreaks. The pathways are illustrated in the directed acyclic graph (DAG) (Supplementary Appendix Fig. S1). Agencies conducting more frequent routine inspections may differ in grading and disclosure policies that affect the inherent foodborne illness outbreak preventive effects.

Agencies requiring inspection grade disclosure may receive more complaints from the public who may be more motivated to report an illness when equipped with the knowledge of an undesirable grade. Therefore, the number of complaints can be considered a mediator in the causal pathway between grading and disclosure and reported outbreaks. Following receipt of consumer illness complaints, agencies operating a centralized complaint database may differ in the number of reported restaurant outbreaks from those without such a database. Additionally, those collecting food history data may also have different capabilities to identify and report restaurant outbreaks from consumer complaints.

Predictors included in the model were either agency-level restaurant grading and disclosure practices or operational characteristics of consumer complaint surveillance. The prevention predictors included the following: disclosure (routinely disclosed restaurant inspection results to the public = 1 and did not disclose = 0), grading (used summary grades to categorize restaurant inspection results = 1 and did not grade = 0), numerical score (used a numerical score for grading restaurant inspection results = 1 and did not use = 0), letter grade (used a letter grade for grading restaurant inspection results = 1 and did not use = 0), and the average annual number of routine restaurant inspections performed by the agency.

The surveillance predictors included collection of food history data, usually of 3 days or more, from consumers complaining about a possible foodborne illness (collected any food history = 1 and no food history collected = 0); operation of a centralized database to allow for analysis and detection of clusters of complaints associated with food service establishments or repeated complaints regarding the same establishment (used a centralized database = 1 and did not use = 0); and the 3-year average number of complaints received by the agency.

We also included, as a model offset, the number of food establishments to account for differences in jurisdiction size. We used Pearson correlation coefficients to assess multicollinearity between predictors. We found a moderate association only between letter grading and point-of-service disclosure (correlation coefficient = 0.461; *p* = 0.0060). Based on our DAG and because these were our primary predictors of interest, we kept the grading and disclosure methods in the final model.

## Results

One hundred forty restaurant inspection agencies responded to the Restaurant Grading Survey (response rate = 17.7%). We contacted those 140 respondents and received 77 local inspection agency responses (55% response rate). Most responding agencies represented county government-level agencies, followed by city and city–county combination agencies (Table 1). Most respondents (91%) reported having a consumer complaint surveillance system that allows the public to report suspected foodborne illnesses that might have been caused by a restaurant.

**Table 1.** Summary Statistics of Local Agency Respondents to the Follow-Up Restaurant Grading Survey Focused on Complaint Systems, 2016–2018 (*N* = 77)

	*n *(%)
Agency type	
County	50 (65)
City	10 (14)
City–county combination	9 (12)
District	5 (6)
Other, specify	3 (4)
Regional collaboration	1
Town	1
Five County Public Health Agency	1
Does your agency have a consumer complaint surveillance system that allows the public to report foodborne illnesses they suspect were caused by particular products, events, or food establishments?	
No	7 (9)
Complaints are taken by the state health department or other state agency	3 (43)
Too few complaints received to warrant a complaint system	1 (14)
Lack of personnel	2 (29)
Lack of resources	3 (43)
Complaint surveillance is not effective in detecting foodborne illness outbreaks	1 (14)
Yes	70 (91)
Statewide phone number	14 (20)
Local phone number	68 (97)
E-mail	59 (84)
Online complaint form	47 (67)
Our agency monitors social media (e.g., health department Twitter)	18 (26)
Privately managed reporting site reports to our agency (e.g., iwaspoisoned.com)	16 (23)
Other, specify:	4
In person (face to face)	2
311 System	1
State Department of Health website	1
Do you have a complaint system in which data are collected into one database that can be analyzed to look for clusters of complaints or repeated complaints regarding the same facility (centralized database)?	70
Yes	47 (67)
No	16 (23)
Other	7 (10)
How do complaints get recorded by your agency?	
Informally on an available sheet of paper	4 (6)
E-mail inbox	8 (11)
Paper log	12 (17)
Computer log: local hard drive	17 (24)
Computer log: noncloud-based database accessible by multiple computers	19 (27)
Computer log: cloud-based database	40 (57)
Other	2 (3)
Upon initial intake of a complaint, what information is recorded by your agency?	70
Contact information of the caller	70 (100)
Food eaten by the complainant at the complaint location	64 (91)
Illness symptoms	67 (96)
Location/establishment of complaint	69 (99)
Medical diagnosis if health care was sought	56 (80)
Number of ill persons who ate food from the complaint location	59 (84)
Suspected food product and product packaging information (if applicable)	58 (83)
Stool specimen was obtained by the health care provider	30 (43)
Willing to give a stool specimen to public health	23 (33)
Food history	52 (74)
3-Day food history	43
5-Day food history	4
>5-Day food history	5
Date of illness onset	61 (87)
Time of illness onset	60 (86)
Date of illness recovery	50 (71)
Time of illness recovery	44 (63)
Other potentially relevant nonfood exposures	43 (61)
Other	4 (6)

Those that did not have a system (*n* = 7, 9%) cited that complaints were taken by the state health department or other state agency (*n* = 3), they lacked resources (*n* = 3) or personnel (*n* = 2) to operate a system, or they had too few complaints to warrant a system (*n* = 1). We limited further analysis to those agencies with a complaint system.

Of the 70 agencies that had some system for collecting consumer complaints, 47 (67%) had a centralized system in which data were collected into one database that could be analyzed to look for clusters. Those who chose “Other” (*n* = 7) explained that they were in the process of developing a system and were therefore recategorized as not having a centralized system. These responses were combined with those who answered that they did not have a centralized complaint system (total *n* = 23).

Although recommended by the Council to Improve Foodborne Outbreak Response (2019), only 52 (74%) agencies routinely collected any food history from complainants. Forty-three agencies collected a 3-day food history, while nine agencies collected food history data of five or more days (Table 1).

### Surveillance factors only provide partial understanding of the number of outbreaks reported

A zero-truncated negative binomial regression model showed that the two continuous variables of average number of complaints received (IRR = 1.001, 95% confidence interval [CI] = 1.0004–1.002, *p* = 0.005) and average annual number of routine inspections performed (IRR = 0.99994, 95% CI = 0.9999–0.99999, *p* = 0.013) were statistically significantly associated with the number of restaurant outbreaks received.

Overall, the model had a statistically significant goodness of fit, as measured by the LRT (LRT *p* = 0.025) (Table 2). After adjusting for the surveillance predictors, collecting food history data and operating a centralized complaint system, the average number of complaints (IRR = 1.0006, 95% CI = 0.9997–1.002, *p* = 0.190) and the overall model were no longer statistically significant in predicting the number of restaurant outbreaks (LRT *p* = 0.09) (Table 3).

**Table 2.** Zero-Truncated Negative Binomial Regression Analysis of Restaurant Outbreaks by Complaint and Inspection Rates

	IRR	Standard error	95% Confidence interval	*p*
Avg. No. of complaints	1.001	0.0005	1.0004–1.002	0.005
Avg. No. of routine inspections	0.99994	0.00002	0.9999–0.99999	0.013
Intercept	0.0012	0.0005	0.0006–0.0026	<0.001

Likelihood ratio test *p* = 0.025.

Avg. No., average number; IRR, incidence rate ratio.

**Table 3.** Zero-Truncated Negative Binomial Regression Analysis of Restaurant Outbreaks by Surveillance Factors and Average Number of Complaints Received

	IRR	Standard error	95% Confidence interval	*p*
Any food history	3.35	2.68	0.697–16.10	0.131
Centralized database	1.89	1.09	0.615–5.82	0.266
Avg. No. of complaints	1.0006	0.0005	0.9997–1.002	0.190
Intercept	0.0002	0.0002	0.00003–0.0016	<0.001

Likelihood ratio test *p* = 0.09.

IRR, incidence rate ratio.

### Prevention factors alone only provide partial understanding of the relationship between grading and disclosure practices and the number of outbreaks reported

The zero-truncated negative binomial regression model with prevention factors as predictors of the number of restaurant outbreaks showed only one statistically significant predictor (letter grade) (IRR = 0.056, 95% CI = 0.006–0.55, *p* = 0.013), but the overall model did not have a statistically significant goodness of fit (LRT *p* = 0.206) (Table 4). In this model, the average number of routine inspections performed per year was no longer significant.

**Table 4.** Zero-Truncated Negative Binomial Regression Analysis of Restaurant Outbreaks by Prevention Factors

	IRR	Standard error	95% Confidence interval	*p*
Online	0.436	0.343	0.093–2.04	0.291
Point of service	2.11	1.97	0.34–13.14	0.423
Numerical score	1.39	0.928	0.38–5.14	0.617
Letter grade	0.056	0.065	0.006–0.55	0.013
Avg. No. of routine inspections	1.000	0.00003	0.9999–1.00	0.872
Intercept	0.0028	0.0019	0.00075–0.010	<0.001

Likelihood ratio test *p* = 0.206.

IRR, incidence rate ratio.

As supported by the statistically significant LRT (*p* = 0.0015), we chose the final model (Table 5) that combines prevention and surveillance factors rather than the individual models that only include one set of factors or the other. Agencies operating a centralized complaint database reported twice as many (IRR = 2.03, 95% CI = 1.06–3.91, *p* = 0.033) outbreaks than those without a centralized database when controlling for surveillance and other prevention factors in a fully adjusted model.

**Table 5.** Fully Adjusted Zero-Truncated Negative Binomial Regression Analysis of Restaurant Outbreaks, 2016–2018

	IRR	Standard error	95% Confidence interval	*p*
Online	0.76	0.361	0.303–1.93	0.569
Point of service	1.20	0.532	0.502–2.86	0.683
Numerical score	2.92	1.16	1.33–6.38	0.007
Letter grade	0.074	0.052	0.186–0.295	<0.001
Any food history	2.50	2.26	0.43–14.7	0.309
Centralized database	2.03	0.68	1.06–3.91	0.033
Avg. No. of complaints	1.001	0.0004	1.0006–1.002	<0.001
Avg. No. of routine inspections	0.99995	0.00002	0.99990–0.999998	0.039
Intercept	0.0004	0.0003	0.0001–0.0016	<0.001

Likelihood ratio test *p* = 0.0015.

IRR, incidence rate ratio.

The number of restaurant outbreaks reported by agencies that used numerical scoring was three times (IRR = 2.92, 95% CI = 1.33–6.38, *p* = 0.007) the outbreaks reported by those that did not use numerical scores. Agencies using letter grading reported 93% fewer (IRR = 0.074, 95% CI = 0.186–0.295, *p* = <0.001) outbreaks than did those not using letter grades. These two most frequently used grading methods were included in the model as nonmutually exclusive categories.

Compared with Table 4, the estimate for agencies that disclosed inspection results to the public through an online platform increased to 0.76 from 0.436 (IRR = 0.76, 95% CI = 0.303–1.93, *p* = 0.569), while the estimate for those disclosing at the point of service decreased to 1.20 from 2.11 (IRR = 1.20, 95% CI = 0.502–2.86, *p* = 0.683); however, these estimates were not statistically significant.

For food history, those that did collect these data reported 2.5 times more outbreaks (IRR = 2.50, 95% CI = 0.43–14.7, *p* = 0.309) compared with those that did not collect food history data (Table 5).

## Discussion

Routine restaurant inspection and inspection grading and disclosure provide some understanding of prevention effectiveness. Disclosure and grading practices are intended to motivate restaurant operators to prioritize food safety within their establishments. After adjusting for surveillance factors, grading using a letter grade significantly prevented outbreaks in restaurant settings. However, the numerical score was associated with increased outbreaks, potentially due to increased complexity in the interpretation of numerical scores compared with letter grades. The preventive effects of grading rely heavily on consumers' ability to interpret the grade or score.

Letter grading could be more easily understandable by the public than numerical scores, where a difference in a few points may have a negligible effect on consumers' decision to dine at a certain restaurant. The methods used to disclose these grades to the public warrant further investigation as disclosure factors were not statistically significant in the prevention-specific and fully adjusted models.

Due to the low number of respondents in our Complaint System Survey, our analysis was underpowered to detect statistically significant differences in outbreak outcomes among prevention and surveillance factors. Future studies with sufficient sample size may be useful to determine the pathogen-stratified effects of prevention and surveillance measures.

Our findings also showed the importance of routine restaurant inspections as a prevention measure, even after adjusting for differences in surveillance methods that could hinder detection and reporting.

### Centralized surveillance database systems are useful in detecting outbreaks

Centralized systems allow for detection of outbreak signals that occur over time. This is essential for pathogens with longer incubation periods, such as *Salmonella* and Shiga toxin–producing *E. coli* (Hedberg et al., 2008). Among respondents to our survey, public health outcomes for those without a complaint system were limited, which could be an indication of limited ability to detect outbreaks. Toxin-mediated outbreaks are often related to single-occurrence events and therefore may be detected by a single complaint report.

Outbreak detection from a single complaint report may not reflect the full utilization of a centralized database. Consistent with a previous study documenting the improved ability of centralized databases to detect outbreaks (Li et al., 2011), we concluded that having a centralized database to manage consumer complaints is useful for detecting more outbreaks than not having a centralized database.

### Increasing complaints can detect more outbreaks

The association between increased complaint rates and increased ability to detect outbreaks found in this study is consistent with previous findings (Kim et al., 2022). Complaint quality should also be considered by agencies looking to increase their complaint intake volume. Collecting food history data reduces the chance for last meal bias in reporting, and management of these complaint data in a central location allows for analysis to detect outbreak signals.

Although not statistically significant, our results suggest that collecting food history of 3 days or more was a practice that may detect more outbreaks than not collecting this information.

### Limitations

It has been estimated that 22% of small agencies with jurisdictions of less than 250,000 people do not have the capacity to record and respond to foodborne illness complaints (National Environmental Health Association, 2013). It is possible that unmeasured agency-specific reporting rules and capacity factors such as staffing characteristics, including full-time equivalent levels and training, can affect complaint system management and outbreak investigation and reporting.

Because the study sample was a subset of respondents to a previously conducted survey, it is worth noting that a larger sample size may have produced more significant and robust associations. The original Restaurant Grading Survey used a respondent pool of agencies participating in the U.S. Food and Drug Administration Voluntary National Retail Food Regulatory Program Standards program. This limits our ability to generalize the results of this analysis to agencies beyond those participating in this program.

Because few states were included in the sensitivity analysis due to missing data in our dataset, assessment of clustering by state using a different dataset may be needed.

## Conclusions

Studies on the effects of public health practice often examine the effects of prevention and surveillance measures separately, although these measures work simultaneously in practice. When we examined these factors separately, results were inconsistent in predicting the effects they had on the number of outbreaks. However, when combined in a fully adjusted model, the prevention and surveillance factors predicted the outcome in a cohesive and significant manner.

The novel framework introduced in this study will be a powerful tool for future evaluations of complaint-based surveillance systems that account for any biases introduced by effective prevention measures. Improving consumer complaint system structure and management can bolster outbreak detection and maximize limited public health resources while increasing the efficiency of complaint-based surveillance.

## Supplementary Material

Supplemental data
