# Supplementary figures and images for: Foodborne Illness Complaint Systems Detect, and Restaurant Inspection Programs Prevent Restaurant-Associated Foodborne Illness Outbreaks
Source: Foodborne Pathog Dis. 2024 Feb 5;21(2):92–8. doi: 10.1089/fpd.2023.0086 (PMC10877379; doi:10.1089/fpd.2023.0086)

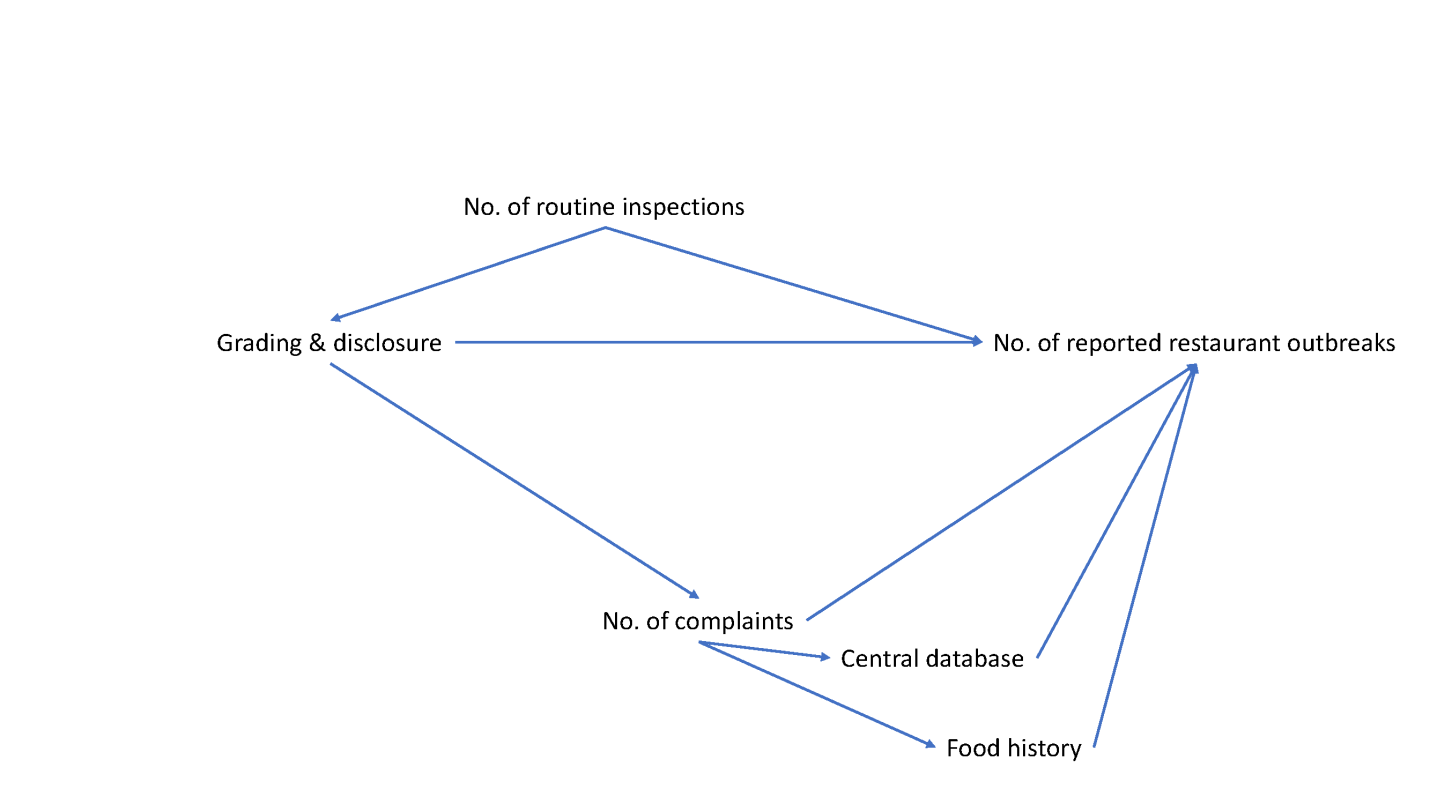

Supplement: Supplemental data [file Suppl_Appendix-Figure-S1.docx]
